# High Chloride Burden and Clinical Outcomes in Critically Ill Patients With Large Hemispheric Infarction

**DOI:** 10.3389/fneur.2021.604686

**Published:** 2021-05-20

**Authors:** Dong-Seok Gwak, Inyoung Chung, Baik-Kyun Kim, Sukyoon Lee, Han-Gil Jeong, Yong Soo Kim, Heeyun Chae, Chan-Young Park, Moon-Ku Han

**Affiliations:** ^1^Department of Neurology, Kyungpook National University Hospital, Daegu, South Korea; ^2^Department of Neurology, Nowon Eulji Medical Center, Seoul, South Korea; ^3^Department of Neurology, Chungbuk National University Hospital, Cheongju-si, South Korea; ^4^Department of Neurology, Inje University Busan Paik Hospital, Busan, South Korea; ^5^Division of Neurocritical Care, Department of Neurosurgery and Neurology, Seoul National University Bundang Hospital, Seongnam, South Korea; ^6^Department of Neurology, Seoul National University Bundang Hospital, Seongnam, South Korea; ^7^Department of Neurology, Seoul National University College of Medicine, Seoul, South Korea

**Keywords:** chloride, critical care, cerebral infarction, mortality, acute kidney injury, brain edema

## Abstract

**Background:** In general, disease severity has been found to be associated with abnormal chloride levels in critically ill patients, but hyperchloremia is associated with mixed results regarding patient-centered clinical outcomes. We aimed to investigate the impact of maximum serum chloride concentration on the clinical outcomes of critically ill patients with large hemispheric infarction (LHI).

**Methods:** We conducted a retrospective observational cohort study using prospective institutional neurocritical care registry data from 2013 to 2018. Patients with LHIs involving over two-thirds of middle cerebral artery territory, with or without infarction of other vascular territories, and a baseline National Institutes of Health Stroke Scale score of ≥13 were assessed. Those with a baseline creatinine clearance of <15 mL/min and required neurocritical care for <72 h were excluded. Primary outcome was in-hospital mortality. Secondary outcomes included 3-month mortality and acute kidney injury (AKI) occurrence. Outcomes were compared to different maximum serum chloride levels (5 mmol/L increases) during the entire hospitalization period using multivariable logistic regression analyses.

**Results:** Of 90 patients, 20 (22.2%) died in-hospital. Patients who died in-hospital had significantly higher maximum serum chloride levels than did those who survived up to hospital discharge (139.7 ± 8.1 vs. 119.1 ± 10.4 mmol/L; *p* < 0.001). After adjusting for age, sex, and Glasgow coma scale score, each 5-mmol/L increase in maximum serum chloride concentration was independently associated with an increased risk of in-hospital mortality (adjusted odds ratio (aOR), 4.34; 95% confidence interval [CI], 1.98–9.50; *p* < 0.001). Maximum serum chloride level was also an independent risk factor for 3-month mortality (aOR, 1.99 [per 5 mmol/L increase]; 95% CI, 1.42–2.79; *p* < 0.001) and AKI occurrence (aOR, 1.57 [per 5 mmol/L increase]; 95% CI, 1.18–2.08; *p* = 0.002).

**Conclusions:** High maximum serum chloride concentrations were associated with poor clinical outcomes in critically ill patients with LHI. This study highlights the importance of monitoring serum chloride levels and avoiding hyperchloremia in this patient population.

## Introduction

Large hemispheric infarction (LHI) is a life-threatening condition that affects the entirety or majority of the middle cerebral artery territory, with or without anterior and posterior cerebral artery involvement, and has a mortality rate of 40–80% in untreated LHI patients (1). Cerebral edema occurs in LHI patients, which often leads to increased intracranial pressure, transtentorial herniation, and neurological deterioration (2). Because of the propensity to cause or exacerbate brain edema by hypo-osmolar balanced crystalloids, normal saline (0.9% NaCl solution) is more frequently used and hypertonic saline is commonly used for the management of cerebral edema (3). However, large volume of both normal saline and hypertonic saline may raise the risk of hyperchloremia (4–7).

Hyperchloremia has been reported to be associated with high hospital mortality and poor outcome in general critically ill patients (8–13) and can lead to metabolic acidosis (14), has negative effects on renal blood flow (15), and is associated with acute kidney injury (AKI) development (16–18). Previous studies have shown that hyperchloremia is associated with high rates of in-hospital mortality in patients with subarachnoid hemorrhages (19), intracerebral hemorrhages (20), and traumatic brain injuries (21).

However, whether hyperchloremia may be associated with hospital mortality in patients with LHI remains largely unknown. In this study, we sought to evaluate the impacts of serum chloride level on the clinical outcomes of critically ill patients with LHI.

## Materials and Methods

### Study Design and Population

**Figure 1:**
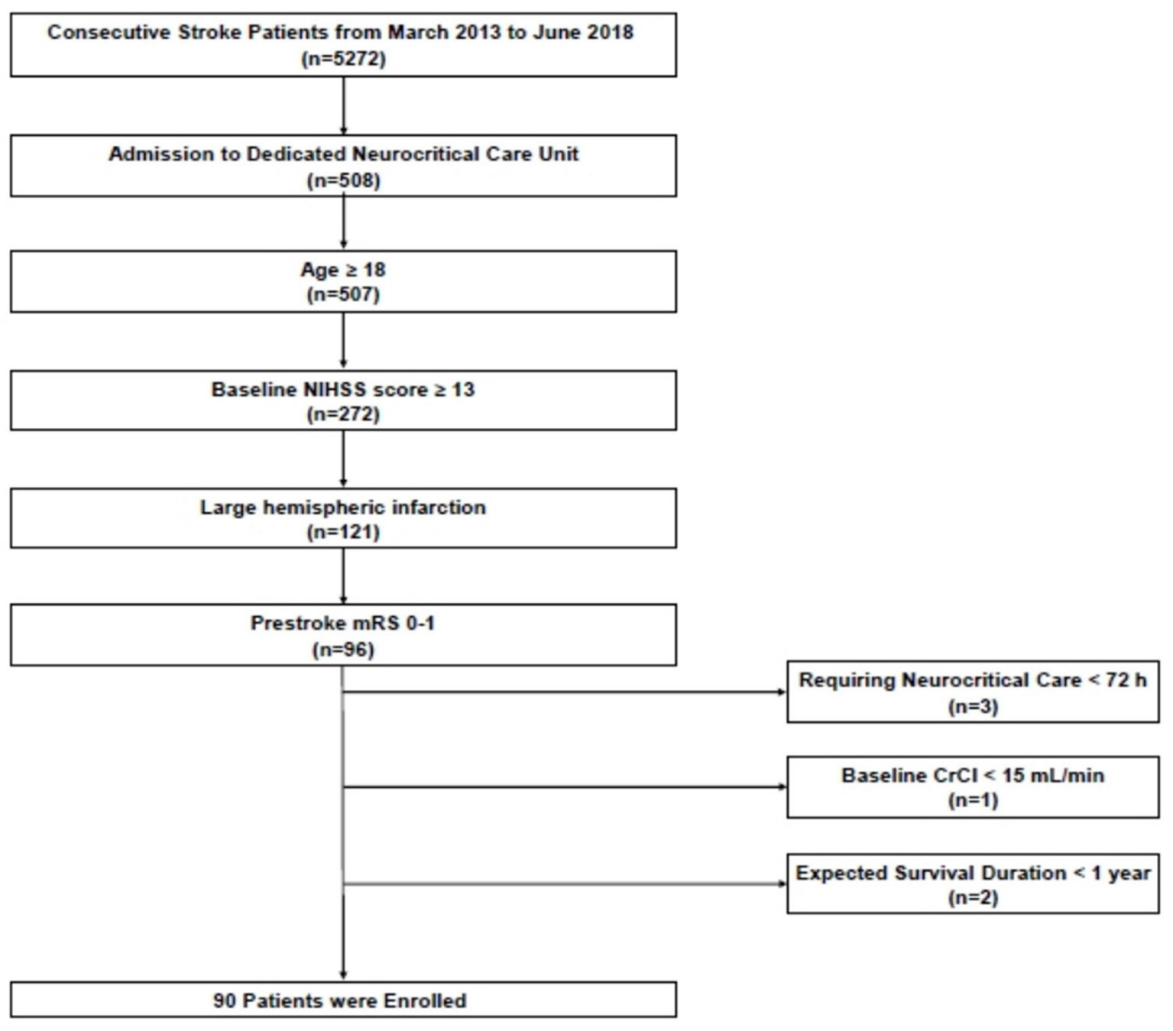
Flowchart of patient screening and enrollment. NIHSS, National Institutes of Health Stroke Scale.

We retrospectively analyzed patient data from a prospective institutional neurocritical care registry. Among 5,272 consecutive ischemic stroke patients who were admitted to the Seoul National University Bundang Hospital within 7 days after symptom onset between March 2013 and June 2018, we identified patients who fulfilled the following inclusion criteria: (1) admission to a dedicated neurocritical care unit (*n* = 508), (2) 18 years of age or older (*n* = 507), (3) National Institutes of Health Stroke Scale (NIHSS) score ≥ 13 at arrival (*n* = 272), (4) LHI involving over two-thirds of middle cerebral artery territory, with or without infarction of other vascular territories (*n* = 121), and (5) pre-stroke modified Rankin Scale (mRS) score of 0–1 (*n* = 96). Patients were excluded from this study if they required neurocritical care for <72 h (*n* = 3) and had a creatinine clearance (estimated using the Cockcroft and Gault equation) of <15 mL/min or end-stage renal disease requiring dialysis before admission (*n* = 1). Those with an expected survival duration of <1 year (*n* = 2) were also excluded (Figure 1). The primary objective of this study was to determine whether the maximum serum chloride level over the entire duration of hospitalization was associated with in-hospital mortality. Our secondary objective was to assess the association between maximum serum chloride levels and 3-month mortality and AKI occurrence. Our study proposal was approved by the institutional review board (IRB approval number: B-1908/556-105). Informed consent was waived because of the retrospective nature of the study, and all study participants were fully de-identified.

### Data Collection

Data on baseline demographic and clinical characteristics that may affect patients' clinical outcomes were obtained from the prospective institutional neurocritical care registry database or through the electronic chart review method. Data on the following variables were collected: age; height; weight; sex; pre-stroke mRS score; onset-to-arrival time; baseline NIHSS score; Glasgow Coma Scale (GCS) score with the worst value at the admission day of neurocritical care unit; systolic and diastolic blood pressure; oxygen requirement (PaO_2_/FiO_2_); previous history of stroke, hypertension, diabetes mellitus, hyperlipidemia, smoking, atrial fibrillation, and anemia; treatment variables patients received, including type of recanalization therapy; mannitol, hypertonic saline (11.7% NaCl), and its total dose administrated, fluid balance defined by fluid intake—output, nephrotoxic agents, antibiotics, contrast media; targeted temperature management (TTM), decompressive surgery, renal replacement therapy (RRT), and mechanical ventilation; pneumonia, length of stay in the neurocritical care unit, and duration of hospitalization; imaging variables including initial and maximum infarct volume evaluated by the ABC/2 method (22), and maximum midline shift measured at the level of septum pellucidum (23); and laboratory data, including baseline and maximum serum chloride, sodium, and creatinine levels, baseline and minimum serum bicarbonate levels, maximum base deficit, blood urea nitrogen levels, baseline creatinine clearance (assessed using the Cockcroft and Gault equation), AKI development based on Kidney Disease: Improving Global Outcome (KDIGO) criteria (24), hemoglobin levels, and baseline blood glucose level.

### Outcomes

The primary outcome was in-hospital mortality. Secondary outcomes included 3-month mortality and occurrence of any AKI (KDIGO Stage ≥ 1) and moderate to severe AKI (KDIGO stage ≥ 2). AKI stage 1 represents a ≥ 0.3-mg/dl increase in serum creatinine levels over a 48-h period, a 1.5-fold to 2-fold increase from baseline serum creatinine levels, or a urine output of ≤ 0.5 ml/kg/h for more than 6 h. AKI stage 2 was defined as a 2-fold to 3-fold increase from baseline serum creatine levels or a urine output of ≤ 0.5 ml/kg/h for more than 12 h. AKI stage 3 represents a > 3-fold increase in serum creatinine levels from baseline (or a serum creatinine level of ≥ 4.0 mg/dl with an acute increment of at least 0.5 mg/dl), the need for initiating RRT, a urine output of ≤ 0.3 ml/kg/h for 24 h, or a 12-h period of anuria (24).

### Statistical Analysis

Bivariate analyses between patients who died in-hospital and those who survived up to hospital discharge were performed using Pearson's chi-square test, Fisher's exact method for categorical variables, Student's *t*-test, or the Mann–Whitney *U*-test for continuous variables, as appropriate. Data are expressed as number (%), mean ± standard deviation, or median (interquartile range [IQR]).

Next, we performed univariable logistic regression analyses to assess the association between the maximum serum chloride level during the entire period of hospitalization and in-hospital mortality. Because of the small sample size, we tested several multivariable logistic models with prespecified covariates. Model 1 adjusted for demographics (age and sex) and the well-known predictor of disease severity (GCS score). Model 2 adjusted for imaging variables regarding infarct and edema volumes (initial infarct volume, maximum infarct volume, and midline shift). Model 3 adjusted for well-known risk factors for mortality in critically ill patients (fluid balance and mechanical ventilation). Model 4 adjusted for covariates with a *p* < 0.1 in the bivariate analysis with the forward stepwise selection method. Model 5 adjusted for hyperchloremia, hypernatremia, age, sex, and GCS score. The cutoff values of hyperchloremia and hypernatremia were determined by the point maximizing Youden's index (sensitivity + specificity −1) in receiver operating characteristic curves (25). The associations of maximum chloride levels with 3-month mortality were tested with the same models. Those who had missing mortality data at 3 months (*n* = 2) were excluded from the corresponding analysis. Moreover, logistic regression analyses for determining the risk factors for AKI were performed using a forward stepwise selection method among imbalanced variables (*p* < 0.10) between the groups with and without AKI. A two-sided *p* < 0.05 was considered statistically significant. All statistical analyses were tested with SPSS version 25.0 for Windows.

## Results

A total of 90 LHI patients were enrolled in this study. Of these, 20 (22.2%) patients died in-hospital. Patients' baseline characteristics are shown in Table 1. Those who died during hospitalization had significantly lower GCS scores (3.0 [IQR 3.0–5.0] vs. 6.5 [3.0–9.0]; *p* = 0.002), higher systolic blood pressure (158.7 vs. 150.2; *p* = 0.048), and more hyperlipidemia (50.0 vs. 25.7%, *p* = 0.039) and received treatment *via* hypertonic saline (95.0 vs. 48.6%; *p* < 0.001), TTM (75.0 vs. 41.4%; *p* = 0.008), and mechanical ventilation (90.0 vs. 61.4%; *p* = 0.016) more frequently than the survivors.

**Table 1:** Patients' baseline demographic and clinical characteristics.

**Characteristics**	**Total**	**Deceased**	**Survived**	***P*-value**
	**(*N* = 90)**	**(*N* = 20)**	**(*N* = 70)**	
Age, years	71.0 ± 12.6	72.7 ± 10.6	70.6 ± 13.1	0.869
Height, cm	162.7 ± 9.1	160.3 ± 9.2	163.4 ± 9.0	0.163
Weight, kg	61.5 ± 11.0	59.7 ± 8.0	62.0 ± 11.8	0.412
Male	50 (55.6%)	10 (50.0%)	40 (57.1)	0.571
Pre-stroke mRS score				1.000
0	78 (86.7)	18 (90.0)	60 (85.7)	
1	12 (13.3)	2 (10.0)	10 (14.3)	
Onset-to-arrival time, h	1.7 (0.8–7.0)	1.4 (0.8–4.4)	1.7 (0.9–8.3)	0.491
NIHSS score	19.7 ± 4.4	19.7 ± 4.2	19.7 ± 4.6	0.853
GCS score	5.0 (3.0–8.3)	3.0 (3.0–5.0)	6.5 (3.0–9.0)	0.002
SBP, mmHg	152.1 ± 26.1	158.7 ± 20.6	150.2 ± 27.3	0.048
DBP, mmHg	83.2 ± 16.3	88.8 ± 12.9	81.6 ± 16.9	0.087
PaO2/FiO2 ≤ 300	30 (33.3)	7 (35.0)	23 (32.9)	0.858
**Past medical history**				
Hypertension	59 (65.6)	14 (70.0)	45 (64.3)	0.635
Diabetes mellitus	22 (24.4)	4 (20.0)	18 (25.7)	0.771
Hyperlipidemia	28 (31.1)	10 (50.0)	18 (25.7)	0.039
Smoking	28 (31.1)	3 (15.0)	25 (35.7)	0.078
Atrial fibrillation	53 (58.9)	15 (75.0)	38 (54.3)	0.097
Previous stroke	16 (17.8)	2 (10.0)	14 (20.0)	0.508
Anemia	21 (23.3)	4 (20.0)	17 (24.3)	0.774
**Treatment variables**				
Type of recanalization therapy				0.190
IV tPA	7 (7.8)	3 (15.0)	4 (5.7)	
EVT	24 (26.7)	3 (15.0)	21 (30.0)	
IV tPA + EVT	18 (20.0)	6 (30.0)	12 (17.1)	
Mannitol	58 (64.4)	16 (80.0)	42 (60.0)	0.099
Hypertonic saline	53 (58.9)	19 (95.0)	34 (48.6)	<0.001
Total dose of hypertonic saline, mL	120 (0–660)	360 (135–615)	0 (0–745)	0.053
Fluid balance, mL/kg/d	3.58 (1.48–6.34)	4.41 (0.54–7.51)	3.48 (1.48–6.33)	0.574
TTM	44 (48.9)	15 (75.0)	29 (41.4)	0.008
Decompressive surgery	14 (15.6)	3 (15.0)	11 (15.7)	1.000
RRT	5 (5.6)	3 (15.0)	2 (2.9)	0.071
Nephrotoxic agents	43 (47.8)	8 (40.0)	35 (50.0)	0.430
Antibiotic use	84 (93.3)	19 (95.0)	65 (92.9)	1.000
Contrast media	71 (78.9)	15 (75.0)	56 (80.0)	0.757
Mechanical ventilation	61 (67.8)	18 (90.0)	43 (61.4)	0.016
Pneumonia	66 (73.3)	13 (65.0%)	53 (75.7)	0.339
Length of stay in the ICU, d	10.0 (6.8–18.0)	9.5 (4.3–14.0)	10.0 (7.0–20.0)	0.376
Duration of hospitalization, d	27.5 (14.0–38.0)	11.0 (5.8–14.0)	30.5 (23.0–39.8)	<0.001
**Imaging variables**				
Initial infarct volume, mL	198.0 (170.0–266.3)	246.0 (183.7–295.6)	188.9 (168.2–253.0)	0.045
Maximum infarct volume, mL	297.0 (218.6–390.6)	431.9 (314.3–528.7)	273.6 (207.9–363.3)	<0.001
Maximum midline shift, mm	7.4 (3.7–12.1)	14.7 (12.3–16.8)	5.8 (3.1–9.4)	<0.001

*Values are presented as mean ± standard deviation, median (interquartile range), or number (%)*.

*DBP, diastolic blood pressure; EVT, endovascular therapy; GCS, Glasgow Coma Scale; ICU, intensive care unit; IV tPA, intravenous tissue-type plasminogen activator; mRS, modified Rankin Scale; NIHSS, National Institutes of Health Stroke Scale; RRT, renal replacement therapy; SBP, systolic blood pressure; TTM, targeted temperature management*.

The maximum serum chloride levels (139.7 vs. 119.1 mmol/L; *p* < 0.001), sodium levels (170.9 vs. 152.7 mmol/L; *p* < 0.001), and creatinine levels (2.90 vs. 1.20 mmol/L; *p* < 0.001) were significantly higher, and the minimum value of bicarbonate (15.3 vs. 19.4 mmol/L; *p* < 0.001) was significantly lower in those who died in-hospital (Table 2). However, baseline laboratory findings including serum chloride, sodium, bicarbonate, creatinine, and creatinine clearance levels were not significantly different between the two groups. AKI occurred significantly more frequently in patients who died in-hospital than in those who survived (90.0 vs. 30.0%; *p* < 0.001).

**Table 2:** Laboratory findings and acute kidney injury incidence.

**Laboratory findings**	**Total**	**Deceased**	**Survived**	***P*-value**
	**(*N* = 90)**	**(*N* = 20)**	**(*N* = 70)**	
**Serum chloride levels, mmol/L**				
Baseline	103.2 ± 3.4	102.8 ± 3.2	103.4 ± 3.4	0.399
Maximum	123.7 ± 13.1	139.7 ± 8.1	119.1 ± 10.4	<0.001
**Serum sodium levels, mmol/L**				
Baseline	138.1 ± 2.8	138.4 ± 3.0	138.0 ± 2.8	0.653
Maximum	156.7 ± 11.9	170.9 ± 8.0	152.7 ± 9.5	<0.001
**Serum bicarbonate levels, mmol/L**				
Baseline	22.3 ± 2.4	21.8 ± 1.9	22.5 ± 2.5	0.164
Minimum	18.5 ± 3.1	15.3 ± 2.7	19.4 ± 2.6	<0.001
Maximum base deficit, mmol/L	5.5 ± 3.1	8.7 ± 2.7	4.6 ± 2.6	<0.001
BUN, mg/dL	19.2 ± 9.5	18.8 ± 7.9	19.3 ± 10.0	0.789
**Serum creatinine levels, mmol/L**				
Baseline	0.88 ± 0.38	0.90 ± 0.34	0.88 ± 0.40	0.570
Maximum	1.58 ± 1.15	2.90 ± 1.43	1.20 ± 0.70	<0.001
CrCl*^*a*^*, mL/min	71.3 ± 34.2	64.5 ± 26.5	73.3 ± 36.1	0.470
≥ 90	24 (26.7)	4 (20.0)	20 (28.6)	0.810
60–89	28 (31.1)	8 (40.0)	20 (28.6)	
30–60	33 (36.7)	7 (35.0)	26 (37.1)	
15–30	5 (5.6)	1 (5.0)	4 (5.7)	
AKI incidence	39 (43.3)	18 (90.0)	21 (30.0)	<0.001
Stage 1	14 (15.6)	1 (5.0)	13 (18.6)	
Stage 2	11 (12.2)	7 (35.0)	4 (5.7)	
Stage 3	14 (15.6)	10 (50.0)	4 (5.7)	
Hemoglobin levels, g/dL	13.7 ± 2.0	13.8 ± 1.7	13.6 ± 2.1	0.775
Initial glucose levels, mg/dL	140.5 ± 39.6	159.8 ± 50.3	135.0 ± 34.4	0.033

*Values are presented as mean ± standard deviation or number (%)*.

*AKI, acute kidney injury; BUN, blood urea nitrogen; CrCl, creatinine clearance*.

a *CrCl was calculated using the Cockcroft and Gault equation*.

Since maximum serum creatinine level and RRT had collinearity with AKI occurrence, they were omitted from the multivariable logistic analysis. Minimum serum bicarbonate level was also excluded to avoid multi-collinearity with maximum base deficit. In the univariable logistic analysis, those who had high maximum serum chloride level were associated with high in-hospital mortality (odds ratio [OR] [per 5 mmol/L], 3.42; 95% confidence interval [CI], 1.87–6.26; *p* < 0.001; Table 3 and Supplementary Table 1). The association between maximum serum chloride level and in-hospital mortality remained significant throughout the multivariable logistic models 1–4. Additionally, GCS score in models 1 and 4, mechanical ventilation in model 3, and maximum base deficit in model 4 were independently associated with in-hospital mortality. Because of the multicollinearity between serum maximum chloride and sodium level (variance inflation factor of 18.14), these two variables were dichotomized in model 5 to assess the concomitant effect of hyperchloremia (>132.5 mmol/L) with hypernatremia (>162.5 mmol/L) on mortality. Both hyperchloremia and hypernatremia were significantly associated with in-hospital mortality and so was the GCS score.

**Table 3:** Associations between serum maximum chloride level and in-hospital and 3-month mortality.

**Outcomes**	**Crude** ** OR (95% CI)**	**Adjusted** ** OR (95% CI):** ** Model 1^**a**^**	**Adjusted** ** OR (95% CI):** ** Model 2^**b**^**	**Adjusted** ** OR (95% CI):** ** Model 3^**c**^**	**Adjusted** ** OR (95% CI):** ** Model 4^**d**^**	**Adjusted** ** OR (95% CI):** ** Model 5^**e**^**
In-hospital mortality	3.42 (1.87–6.26)	4.34 (1.98–9.50)	2.77 (1.42–5.40)	3.81 (1.88–7.69)	2.82 (1.38–5.78)	14.05 (2.25–87.94)
3-month mortality	2.01 (1.50–2.70)	1.99 (1.42–2.79)	1.85 (1.32–2.60)	1.97 (1.41–2.76)	1.69 (1.20–2.38)	8.39 (1.34–52.58)

*CI, confidence interval; OR, odds ratio*.

a *Model 1 adjusted for maximum chloride level, age, sex, and Glasgow Coma Scale (GCS) score*.

b *Model 2 adjusted for maximum chloride level, initial infarct volume, maximum infarct volume, and midline shift*.

c *Model 3 adjusted for maximum chloride level, positive fluid balance, and mechanical ventilation*.

d *Model 4 selected variables with forward stepwise selection method among imbalanced variables (p < 0.10) between the survivor and the deceased groups*.

e *Model 5 adjusted for hyperchloremia (> 132.5 mmol/L) hypernatremia (> 162.5 mmol/L), age, sex, and GCS score. The represented OR were the values of hyperchloremia*.

Maximum serum chloride level was significantly related to 3-month mortality (OR, 2.01 [per 5 mmol/L]; 95% CI, 1.50–2.70; *p* < 0.001), even after adjusting for confounding variables in models 1–4 (Table 3 and Supplementary Table 2). In model 5, hyperchloremia, not concomitant hypernatremia, was independently associated with 3-month mortality. Further, high maximum serum chloride level was significantly associated with high risk of any AKI development (OR, 1.35 [per 5 mmol/L]; 95% CI, 1.12–1.64; *p* = 0.002) and remained significant after adjusting for confounders (aOR, 1.57 [per 5 mmol/L]; 95% CI, 1.18–2.08; *p* = 0.002; Table 4). Moreover, it was independently associated with the development of moderate to severe AKI (aOR, 1.98 [per 5 mmol/L]; 95% CI, 1.40–2.81; *p* < 0.001). TTM was also independently associated with the development of any AKI and moderate to severe AKI, and the maximum base deficit was significantly related to any AKI development.

**Table 4:** Logistic regression analyses for determining the risk factors for acute kidney injury.

**Variable**	**Any AKI development**		**Any AKI development**		**Moderate to severe AKI development**	
	**Crude**	***P*-value**	**Adjusted**	***P*-value**	**Adjusted**	***P*-value**
	**OR (95% CI)**		**OR (95% CI)**		**OR (95% CI)**	
[Cl^−^]_max_*^*a*^* (per 5 mmol/L)	1.35 (1.12–1.64)	0.002	1.57 (1.18–2.08)	0.002	1.98 (1.40–2.81)	<0.001
[Na^+^]_max_*^*b*^* (per 5 mmol/L)	1.35 (1.10–1.65)	0.004	–	–	–	–
Hypertonic saline	8.53 (3.03–24.02)	<0.001	–	–	–	–
Mannitol	2.74 (1.08–6.92)	0.033	–	–	–	–
TTM	6.96 (2.73–17.78)	<0.001	3.52 (1.14–10.89)	0.029	5.85 (1.48–23.14)	0.012
Maximum base deficit	1.62 (1.29–2.03)	<0.001	1.27 (0.998–1.60)	0.052	–	–
GCS score	0.72 (0.60–0.86)	<0.001	–	–	–	–
Mechanical ventilation	8.41 (2.61–27.14)	<0.001				
Baseline glucose	1.01 (0.998–1.02)	0.099	–	–	–	–
Hyperlipidemia	2.81 (1.12–7.05)	0.028	–	–	–	–
Maximum infarct volume (per 10 mL)	1.05 (1.01–1.09)	0.016	–	–	–	–
Maximum midline shift (mm)	1.19 (1.08–1.31)	<0.001	–	–	–	–

*AKI, acute kidney injury; CI, confidence interval; GCS, Glasgow Coma Scale; OR, odds ratio; TTM, targeted temperature management*.

a *[Cl^−^]_max_ is defined as the maximum serum chloride concentration during the entire hospitalization period*.

b *[Na^+^]_max_ is defined as the maximum serum sodium concentration during the entire hospitalization period*.

## Discussion

In this study, we found that high maximum serum chloride concentration was associated with increased in-hospital mortality in critically ill LHI patients. Every 5-mmol/L increment in maximum chloride level increases the odds of in-hospital mortality risk by 3.42 (1.87–6.26), and the associations remained significant throughout all logistic models. The maximum serum chloride concentration was also significantly associated with 3-month mortality and AKI development.

Previous studies have reported the associations between hyperchloremia and AKI occurrence and mortality in various patient populations (9–13, 16–21). However, the majority of these studies were conducted with general critically ill populations, and their chloride levels were not as high as those in our study. One study including ischemic stroke and intracerebral hemorrhage patients has suggested a possible association between hyperchloremia and increases in serum chloride level within the first 72 h after admission and poor outcomes, but patients treated with hypertonic saline were excluded from the study (26). Moreover, due to the heterogeneity and varying characteristics of the study populations, it was not possible to generalize this study's results for LHI patients. In our study, we observed that high serum chloride levels during the entire hospitalization were associated with an increased risk of poor clinical outcomes in only ischemic stroke patients.

Several explanations regarding potential detrimental effects of hyperchloremia have been proposed. Hyperchloremic metabolic acidosis can depress myocardial function, resulting in reduced cardiac output and organ hypoperfusion (27). This may be a concern for LHI patients who require adequate cerebral perfusion pressure for preventing additional brain ischemia. Another study has shown that hyperchloremic metabolic acidosis during abdominal aortic aneurysm repair has a negative effect on hemostasis (28). Since the chances of hemorrhagic transformation remarkably increases with the large volume of infarctions and edemas (29, 30), caution may be necessary when administering chloride-liberal fluid to LHI patients. Hyperchloremia also causes renal vasoconstriction by inhibiting renin and angiotensin II secretion, leading to a decrease in glomerular filtration rate and AKI development (31).

Patients treated with TTM were also associated with in-hospital mortality and 3-month mortality, although the associations were not statistically significant after adjusting for confounding variables in logistic model 4. Furthermore, TTM was independently associated with AKI development. However, these results should be interpreted with caution. As treatment strategies were determined at the discretion of attending neurologists, patients expected to have larger maximum cerebral edema may have had a higher chance of receiving TTM. Indeed, patients treated with TTM had a lower GCS score, larger maximum infarct volume, larger maximum midline shift, and more received hyperosmolar therapy than did patients who did not receive TTM. Development of pneumonia was not significantly different between the two groups (Supplementary Table 3). Moreover, the hypothermic state may activate potential neuroprotective mechanisms and TTM has been suggested as one of the treatment options for reducing intracranial pressure due to acute ischemic injury (32–34); furthermore, it is not a traditional risk factor for AKI (35).

In our study cohort, the crude maximum sodium level, a well-known predictor of mortality in neurocritically ill patients, was associated with outcomes (36). When evaluating the association of sodium and chloride in combination with in-hospital mortality, only hyperchloremia was an independent risk factor for in-hospital mortality (Model 4). This does not mean that hypernatremia was not associated with outcome but rather that the estimated regression coefficient can be affected due to the study limitation that the maximum chloride and sodium levels were collinear. Indeed, concomitant hypernatremia with hyperchloremia was also related to in-hospital mortality in model 5. Thus, the results could be interpreted as demonstrating that the maximum chloride level was more strongly associated with in-hospital mortality than with the maximum sodium level in our cohort; external validation should be carried out in future studies.

Given the growing evidence of the deleterious effects of hyperchloremia, new strategies for avoiding chloride accumulation might be considered. Recent large randomized controlled trials demonstrated that chloride-restrictive intravenous fluid administration was associated with lower rates of a composite of new RRT, persistent renal dysfunction, and death from any cause than the use of a chloride-liberal strategy for the critically ill (37). However, it is uncertain whether these results apply to neurocritically ill patients, since the proportion of such patients in this trial was small (17.8%). Furthermore, as hypotonic fluids can exacerbate cerebral edemas through high osmotic pressure gradients between the intravascular and extravascular compartment, they are not traditionally recommended for patients with cerebral edema (1, 38, 39). Hyperosmolar therapy with low-chloride hypertonic solutions might represent another option, as 16.4% hypertonic NaCl/Na-Acetate was associated with a lower rate of AKI development than 23.4% NaCl for the management of cerebral edema in patients with subarachnoid hemorrhage (40). Further research is needed to confirm these treatment strategies.

Our study had several limitations. Due to the study's retrospective observational design, we could not assess all potentially relevant variables, prove causal relationships among variables, and exclude selection bias, especially the bias related to the choice of the type of fluid treatment administered. As such, it may be possible for patients with severe cerebral edema to be treated with more hypertonic saline. Furthermore, although all multivariable models showed significant associations between serum maximum chloride level and outcomes, all confounding variables may not be reliably tested due to the small sample size. Because of the multicollinearity of maximum sodium level with chloride level, the effect of concomitant hypernatremia on outcomes could not be fully examined. Moreover, we were also unable to report the exact cumulative amount of chloride burden after patients underwent fluid infusion.

In conclusion, maximum serum chloride concentration may have an impact on the clinical outcomes of critically ill patients with LHI, specifically in-hospital mortality rate, 3-month mortality rate, and AKI occurrence. Our study highlights the importance of monitoring serum chloride concentration and avoiding hyperchloremia in such patients. Further well-designed studies are required to explore the causal relationship between high chloride burden and clinical outcomes and chloride-restrictive strategies for fluid management.

## Data Availability Statement

The raw data supporting the conclusions of this article will be made available by the authors, without undue reservation.

## Ethics Statement

The studies involving human participants were reviewed and approved by Seoul National University Bundang Hospital (IRB approval number: B-1908/556-105). Written informed consent for participation was not required for this study in accordance with the national legislation and the institutional requirements.

## Author Contributions

D-SG and IC established the study protocol, analyzed and interpreted the data, and wrote the manuscript. B-KK, SYL, H-GJ, YK, HC, and C-YP interpreted the data and revised the manuscript for intellectual content. M-KH established the study idea, interpreted the data, drafted the manuscript, and made critical revisions in the manuscript with intellectual input. All the authors approved the final version of manuscript.

## Conflict of Interest

The authors declare that the research was conducted in the absence of any commercial or financial relationships that could be construed as a potential conflict of interest.
