# Supplementary material for: High Chloride Burden and Clinical Outcomes in Critically Ill Patients With Large Hemispheric Infarction
Source: Front Neurol. 2021 May 20;12:604686. doi: 10.3389/fneur.2021.604686 (PMC8172791; doi:10.3389/fneur.2021.604686)
Supplement: Supplementary file 1 [file Table_1.PDF]

Supplementary Table 1. Logistic regression analysis results for determining risk factors for in-hospital mortality

Variable	Univariable analysis	Multivariable analysis				
	Crude OR (95% CI) *P*-value	Adjusted OR (95% CI) : Model 1[a]	Adjusted OR (95% CI) : Model 2[b]	Adjusted OR (95% CI) : Model 3[c]	Adjusted OR (95% CI) : Model 4[d]	Adjusted OR (95% CI) : Model 5[e]
$[Cl^-]_{max}$[f] (per 5 mmol/L)	3.42 (1.87-6.26)	4.34 (1.98-9.50)	2.77 (1.42-5.40)	3.81 (1.88-7.69)	2.82 (1.38-5.78)	NA
$[Na^+]_{max}$[g] (per 5 mmol/L)	3.18 (1.83-5.50)	NA	NA	NA	NA	NA
Hyperchloremia[h]	36.00 (9.38-138.2)	NA	NA	NA	NA	14.05 (2.25-87.94)
Hypernatremia[i]	36.00 (7.46-173.7)	NA	NA	NA	NA	8.91 (1.18-67.45)
Age	1.01 (0.97-1.06)	1.08 (0.97-1.21)	NA	NA	NA	1.04 (0.95-1.13)
Male	0.75 (0.28-2.03)	3.29 (0.43-25.04)	NA	NA	NA	0.28 (0.05-1.64)
GCS score	0.68 (0.52-0.88)	0.65 (0.43-0.98)	NA	NA	NA	0.65 (0.46-0.93)
Initial infarct volume (per 10 mL)	1.07 (0.996-1.16)	NA	1.09 (0.93-1.27)	NA	NA	NA
Maximum infarct volume (per 10 mL)	1.13 (1.06-1.20)	NA	1.01 (0.90-1.13)	NA	1.14 (1.01-1.29)	NA
Maximum midline shift (per mm)	1.46 (1.23-1.73)	NA	1.19 (0.93-1.51)	NA	NA	NA
Fluid balance (per mL/kg/d)	1.07 (0.99-1.15)	NA	NA	1.14 (0.999-1.30)	NA	NA
Mechanical ventilation	5.65 (1.21-26.31)	NA	NA	6.43 (0.49-83.59)	NA	NA
Maximum base deficit	1.88 (1.39-2.53)	NA	NA	NA	1.70 (1.16-2.50)	NA
Hypertonic saline	20.12 (2.55-158.6)	NA	NA	NA	NA	NA
Mannitol	2.67 (0.81-8.81)	NA	NA	NA	NA	NA
TTM	4.24 (1.39-12.98)	NA	NA	NA	NA	NA
AKI						
Stage 1	1.89 (0.16-22.44)	NA	NA	NA	NA	NA
Stage 2	42.88 (6.59-279.0)	NA	NA	NA	NA	NA

Stage 3	61.25 (9.84-381.3)	NA	NA	NA	NA	NA
Baseline glucose	1.01 (1.002-1.03)	NA	NA	NA	NA	NA
SBP	1.01 (0.993-1.03)	NA	NA	NA	NA	NA
DBP	1.03 (0.996-1.06)	NA	NA	NA	NA	NA
Hyperlipidemia	2.89 (1.03-8.07)	NA	NA	NA	NA	NA
smoking	0.32 (0.09-1.19)	NA	NA	NA	NA	NA
Atrial fibrillation	2.53 (0.83-7.71)	NA	NA	NA	NA	NA

AKI, acute kidney injury; CI, confidence interval; DBP, diastolic blood pressure; GCS, Glasgow coma scale; NA, not applicable; OR, odds ratio; SBP, systolic blood pressure; TTM, targeted temperature management.

[a]Model 1 adjusted for $[Cl^-]_{max}$, age, sex, and GCS score.

[b]Model 2 adjusted for $[Cl^-]_{max}$, initial infarct volume, maximum infarct volume, and midline shift.

[c]Model 3 adjusted for $[Cl^-]_{max}$, positive fluid balance, and mechanical ventilation.

[d]Model 4 selected variables with forward stepwise selection method among imbalanced variables ($p < 0.10$) between the survivor and the deceased groups.

[e]Model 5 adjusted for hyperchloremia, hypernatremia, age, sex, and GCS score

[f]$[Cl^-]_{max}$ is defined as the maximum serum chloride concentration during the entire hospitalization period.

[g]$[Na^+]_{max}$ is defined as the maximum serum sodium concentration during the entire hospitalization period.

[h]Hyperchloremia is defined by value of over 132.5 mmol/L.

[i]Hypernatremia is defined by value of over 162.5 mmol/L/

Supplementary Table 2. Logistic regression analysis results for determining risk factors for three-month mortality

Variable	Univariable analysis	Multivariable analysis				
	Crude OR (95% CI)	Adjusted OR (95% CI) : Model 1[a]	Adjusted OR (95% CI) : Model 2[b]	Adjusted OR (95% CI) : Model 3[c]	Adjusted OR (95% CI) : Model 4[d]	Adjusted OR (95% CI) : Model 5[e]
$[Cl^-]_{max}$[f] (per 5 mmol/L)	2.01 (1.50-2.70)	1.99 (1.42-2.79)	1.85 (1.32-2.60)	1.97 (1.41-2.76)	1.69 (1.20-2.38)	NA
$[Na^+]_{max}$[g] (per 5 mmol/L)	2.06 (1.52-2.79)	NA	NA	NA	NA	NA
Hyperchloremia[h]	14.4 (4.50-46.04)	NA	NA	NA	NA	8.39 (1.34-52.58)
Hypernatremia[i]	9.87 (3.57-27.27)	NA	NA	NA	NA	2.44 (0.48-12.28)
Age	1.02 (0.99-1.06)	1.05 (0.99-1.13)	NA	NA	NA	1.04 (0.98-1.11)
Male	0.48 (0.20-1.17)	1.07 (0.29-3.91)	NA	NA	NA	1.23 (0.34-4.41)
GCS score	0.64 (0.51-0.81)	0.66 (0.50-0.87)	NA	NA	0.73 (0.55-0.97)	0.63 (0.48-0.82)
Initial infarct volume (per 10 mL)	1.04 (0.97-1.11)	NA	0.99 (0.89-1.11)	NA	NA	NA
Maximum infarct volume (per 10 mL)	1.08 (1.03-1.13)	NA	1.01 (0.93-1.11)	NA	NA	NA
Maximum midline shift (per mm)	1.25 (1.13-1.39)	NA	1.05 (0.88-1.24)	NA	NA	NA
fluid balance (per mL/kg/d)	1.05 (0.98-1.12)	NA	NA	1.12 (0.99-1.27)	NA	NA
Mechanical ventilation	13.05 (2.84-59.93)	NA	NA	18.81 (2.06-171.5)	NA	NA
Maximum base deficit	1.81 (1.38-2.38)	NA	NA	NA	1.35 (1.01-1.81)	NA
Hypertonic saline	6.66 (2.24-19.81)	NA	NA	NA	NA	NA
Mannitol	2.68 (0.99-7.22)	NA	NA	NA	NA	NA
TTM	2.69 (1.09-6.65)	NA	NA	NA	NA	NA
AKI						
Stage 1	1.79 (0.40-8.10)	NA	NA	NA	NA	NA
Stage 2	14.33 (3.12-65.96)	NA	NA	NA	NA	NA
Stage 3	32.25 (6.03-172.42)	NA	NA	NA	NA	NA
Baseline glucose	1.02 (1.003-1.03)	NA	NA	NA	NA	NA
SBP	1.01 (0.996-1.03)	NA	NA	NA	NA	NA
DBP	1.03	NA	NA	NA	NA	NA

	(0.998-1.06)					
Hyperlipidemia	2.02 (0.80-5.10)	NA	NA	NA	NA	NA
smoking	0.28 (0.10-0.85)	NA	NA	NA	NA	NA
Atrial fibrillation	1.53 (0.62-3.76)	NA	NA	NA	NA	NA

AKI, acute kidney injury; CI, confidence interval; DBP, diastolic blood pressure; GCS, Glasgow coma scale; NA, not applicable; OR, odds ratio; SBP, systolic blood pressure; TTM, targeted temperature management.

[a]Model 1 adjusted for $[Cl^-]_{max}$, age, sex, and GCS score.

[b]Model 2 adjusted for $[Cl^-]_{max}$, initial infarct volume, maximum infarct volume, and midline shift.

[c]Model 3 adjusted for $[Cl^-]_{max}$, positive fluid balance, and mechanical ventilation.

[d]Model 4 selected variables with forward stepwise selection method among imbalanced variables ($p < 0.10$) between the survivor and the deceased groups.

[e]Model 5 adjusted for hyperchloremia, hypernatremia, age, sex, and GCS score

[f]$[Cl^-]_{max}$ is defined as the maximum serum chloride concentration during the entire hospitalization period.

[g]$[Na^+]_{max}$ is defined as the maximum serum sodium concentration during the entire hospitalization period.

[h]Hyperchloremia is defined by value of over 132.5 mmol/L.

[i]Hypernatremia is defined by value of over 162.5 mmol/L/

Supplementary Table 3. Clinical parameters according to target temperature management.

	Patients treated with TTM (n=44)	Patients not treated with TTM (n=46)	*P*-value
GCS score	3.0 (3.0-6.0)	7.0 (5.0-9.0)	< 0.001
Hypertonic saline	33 (75)	20 (43.5%)	0.002
Mannitol	33 (75)	25 (54.3)	0.041
Maximum infarct volume, mL	345.1 (247.8-419.0)	276.3 (209.9-361.3)	0.047
Maximum midline shift, mm	9.9 ± 5.6	6.4 ± 4.8	0.002
Pneumonia	35 (79.5)	31 (67.4)	0.192

Values are presented as mean ± standard deviation, median (interquartile range), or number (%).

GCS, Glasgow coma scale; TTM, targeted temperature management.
